# Inhibitory Effect of Epigallocatechin Gallate-Silver Nanoparticles and Their Lysozyme Bioconjugates on Biofilm Formation and Cytotoxicity

**DOI:** 10.1021/acsabm.2c00409

**Published:** 2022-08-17

**Authors:** Brahmaiah Meesaragandla, Shahar Hayet, Tamir Fine, Una Janke, Liraz Chai, Mihaela Delcea

**Affiliations:** † Institute of Biochemistry, University of Greifswald, Felix-Hausdorff-Straße 4, 17489 Greifswald, Germany; ‡ ZIK HIKE—Zentrum für Innovationskompetenz “Humorale Immunreaktionen bei kardiovaskulären Erkrankungen”, Fleischmannstraße 42, 17489 Greifswald, Germany; § Institute of Chemistry, The Hebrew University of Jerusalem, Edmond J. Safra Campus, 91904 Jerusalem, Israel; ∥ The Center for Nanoscience and Nanotechnology, The Hebrew University of Jerusalem, Edmond J. Safra Campus, 91904 Jerusalem, Israel; ⊥ DZHK (Deutsches Zentrum für Herz-Kreislauf-Forschung), Partner Site Greifswald, 17489 Greifswald, Germany

**Keywords:** AgNPs, EGCG, human lysozyme, conformational change, antibacterial activity, biofilms

## Abstract

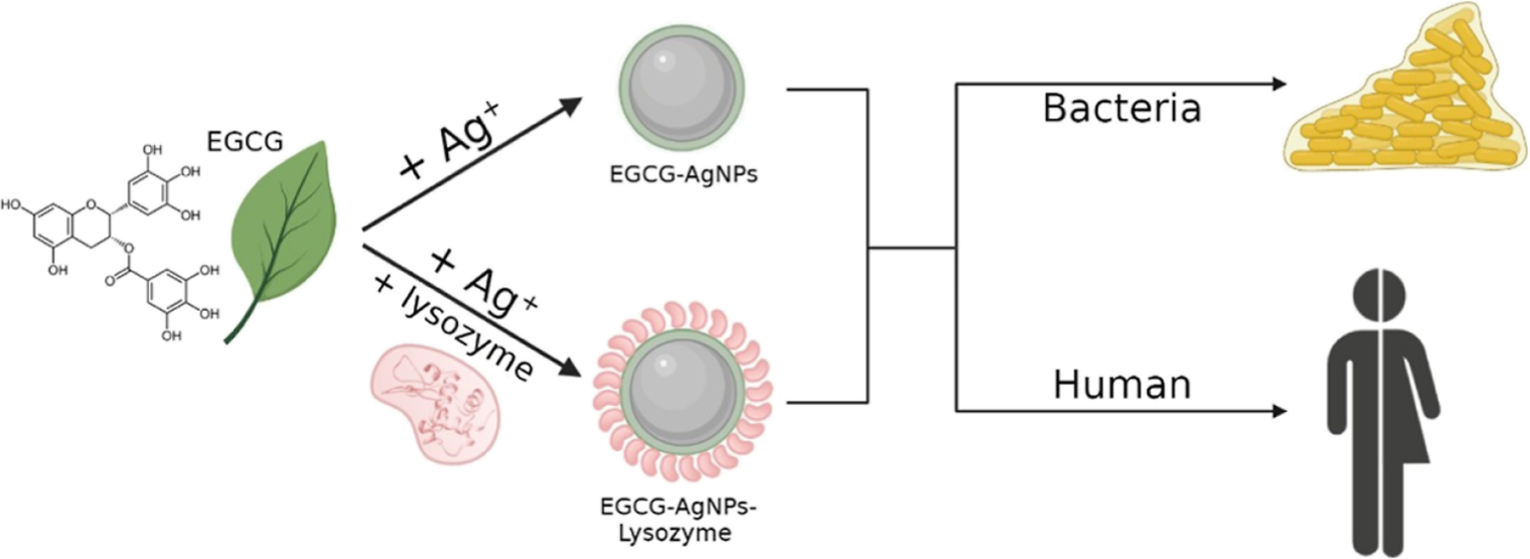

Biofilms are multicellular communities of microbial cells that grow on natural and synthetic surfaces. They have become the major cause for hospital-acquired infections because once they form, they are very difficult to eradicate. Nanotechnology offers means to fight biofilm-associated infections. Here, we report on the synthesis of silver nanoparticles (AgNPs) with the antibacterial ligand epigallocatechin gallate (EGCG) and the formation of a lysozyme protein corona on AgNPs, as shown by UV–vis, dynamic light scattering, and circular dichroism analyses. We further tested the activity of EGCG-AgNPs and their lysozyme bioconjugates on the viability of *Bacillus subtilis* cells and biofilm formation. Our results showed that, although EGCG-AgNPs presented no antibacterial activity on planktonic *B. subtilis* cells, they inhibited *B. subtilis* biofilm formation at concentrations larger than 40 nM, and EGCG-AgNP-lysozyme bioconjugates inhibited biofilms at concentrations above 80 nM. Cytotoxicity assays performed with human cells showed a reverse trend, where EGCG-AgNPs barely affected human cell viability while EGCG-AgNP-lysozyme bioconjugates severely hampered viability. Our results therefore demonstrate that EGCG-AgNPs may be used as noncytotoxic antibiofilm agents.

## Introduction

Biofilms are colonies of bacterial cells that form on surfaces and interfaces. Biofilms may be beneficial, for example, when they develop on plant roots and protect them from pathogens. Detrimental biofilms clog water and oil pipes, and they may lead to death if they infect catheters at hospitals. Cells in a biofilm are held together by an extracellular matrix (ECM) of proteins, polysaccharides, and nucleic acids, which also provides biofilms with mechanical stability and increased antibiotic resistance relative to single cells.^1,2^ In general, antibiotics affect single cells by intervening with cellular growth and proliferation mechanisms, such as cell wall, nucleic acid, or protein synthesis.^3,4^ However, bacterial cells in biofilms are less susceptible to antibiotics compared with free-living cells, partly due to the presence of a protective ECM.^5,6^ Therefore, targeting the ECM formation and/or assembly rather than interfering with cellular proliferation becomes an appealing strategy for biofilm prevention and treatment.^7−10^

Current approaches to prevent biofilm formation include modification of surface topography and surface coatings that detain the cells from sticking to a surface.^11−14^ However, these are temporary treatments because most coatings are quickly covered with self-produced ECM polymers, shielding the antifouling coating and allowing the bacterial cells to stick to the surface, despite the coating. Another method related to biofilm eradication includes the use of biocidal molecules, such as silver nanoparticles (AgNPs).^15,16^ AgNPs comprise one of the most predominant nanomaterials in various products, such as fabrics, bandages, deodorizers, food containers, and disinfectants.^17−20^ AgNPs are also incorporated into hydrogels, creating hybrid antibacterial wound dressings.^21−24^ These applications exploit AgNPs’ excellent optoelectronic properties, such as surface plasmon resonance (SPR), small size, high surface-to-volume ratio, and cost effectiveness.^25,26^ Furthermore, nanosilver is comparatively less reactive than silver ions and is suitable for clinical and therapeutic applications.^27,28^ AgNPs exhibit a broad spectrum of antibacterial and antifungal properties, depending on their shape, size, and surface chemistry.^29−37^ The antibacterial activity of AgNPs is related with the release of Ag^+^ ions that may lead to the formation of radical species, which damage cells to a lethal extent.^38^

Coating the AgNPs with cationic ligands and other antimicrobial molecules can further improve their antimicrobial activity due to synergistic effects.^39,40^ For example, polyhexamethylene biguanide (PHMB)-functionalized AgNPs have shown higher bacteriostatic and bactericidal activity on *Escherichia coli* due to the combined antibacterial effect of AgNPs and PHMB.^41^ Here, we tested the effect of coated AgNPs with epigallocatechin gallate (EGCG) and their lysozyme bioconjugates on biofilm formation. EGCG is a dominant catechin component present in green tea that exhibits various therapeutic properties, including antiobesity, anti-inflammatory, antidiabetic, antitumor effects, and antibacterial activity.^42−45^ EGCG is a strong antioxidant, reported to exhibit anticancer activity against various cancers (e.g., brain, prostate, pancreatic, and bladder) by inducing apoptosis.^46−53^ Additionally, green tea catechins, including EGCG, can kill bacterial cells.^54,55^ It has been proposed that the bactericidal action of EGCG is due to its ability to generate hydrogen peroxide (H_2_O_2_), which damages bacterial membranes.^42,56^ On oral administration of EGCG, the rate of ingestion is less than 5% in humans and below 1% in rats.^57−59^ Such low EGCG bioavailability confines its bioactivity *in vivo*. One way to improve the absorption of EGCG is to adsorb EGCG molecules on AgNPs, which provide a large adsorbent surface per unit mass due to their small size.

As drug delivery agents, nanoparticles can be administered in many ways, including oral, nasal, intraocular, and parenteral. After coming in contact with biological media, nanoparticles can interact with proteins forming a so-called protein corona and may alter protein structure and activity.^60−62^ Among the proteins in the body, lysozyme is an antibacterial enzyme, which catalyzes the hydrolysis of β1,4-glucosidic linkages between *N*-acetylglucosamine and *N*-acetylmuramic acid in the peptidoglycan of the cell wall, particularly in Gram-positive bacteria.^63^ Lysozyme is a small, monomeric, and globular protein, abundant in various body fluids, including serum (4–13 mg/L), saliva, tears, and human milk.^64−66^ It consists of 130 amino acid residues with a molecular mass of 14.7 kDa and belongs to the α + β class of proteins. The compact structure of lysozyme is stabilized by four disulfide bonds, and its surface is typically polar, whereas its inner part is almost hydrophobic. It has been shown that Gram-positive bacteria are more susceptible to the antibacterial activity of lysozyme than Gram-negative bacteria.^67,68^

**Scheme 1:**
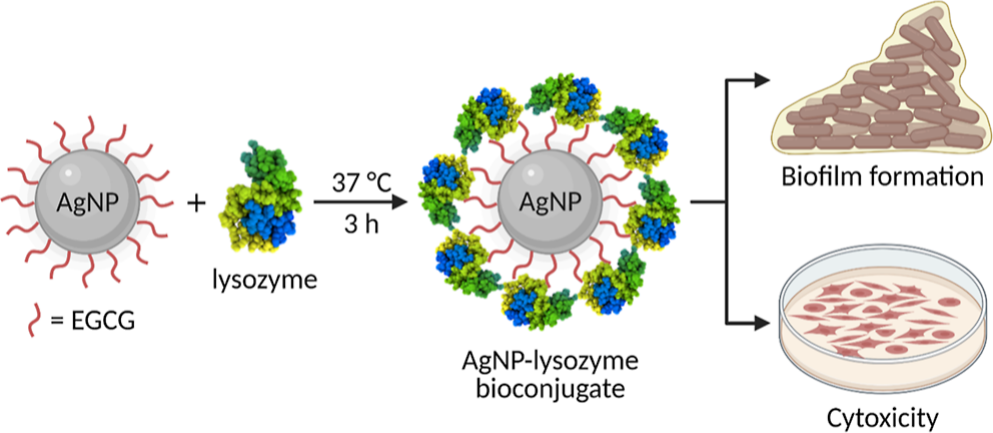
Overview of This Study Illustrating Functionalization of AgNPs with EGCG and the Formation of a Bioconjugate with Lysozyme We have studied the inhibitory effect of EGCG-AgNPs and their lysozyme bioconjugates on biofilm formation and also their cytotoxicity.

In the present study, we have synthesized EGCG-AgNPs to study their antibacterial activity against the Gram-positive wildtype bacterium *Bacillus subtilis* through bacterial growth and biofilm formation assays. To enhance the antibacterial activity of the AgNPs, we also conjugated EGCG-AgNPs with the antibacterial lysozyme (EGCG-AgNP-lysozyme). Indeed, it has been shown that decorating AgNPs with lysozyme and other bactericides may enhance their antibacterial spectrum.^39,40,69^ To attest to the possible application of EGCG-AgNPs and their lysozyme bioconjugates in the human body, we also studied their cytotoxicity effect against human cells (see Scheme 1 for an overview of the study). This study highlights the design of safe and effective EGCG-AgNPs antibiofilm agents.

## Results and Discussion

### Synthesis and Characterization of EGCG-AgNPs

**Figure 1:**
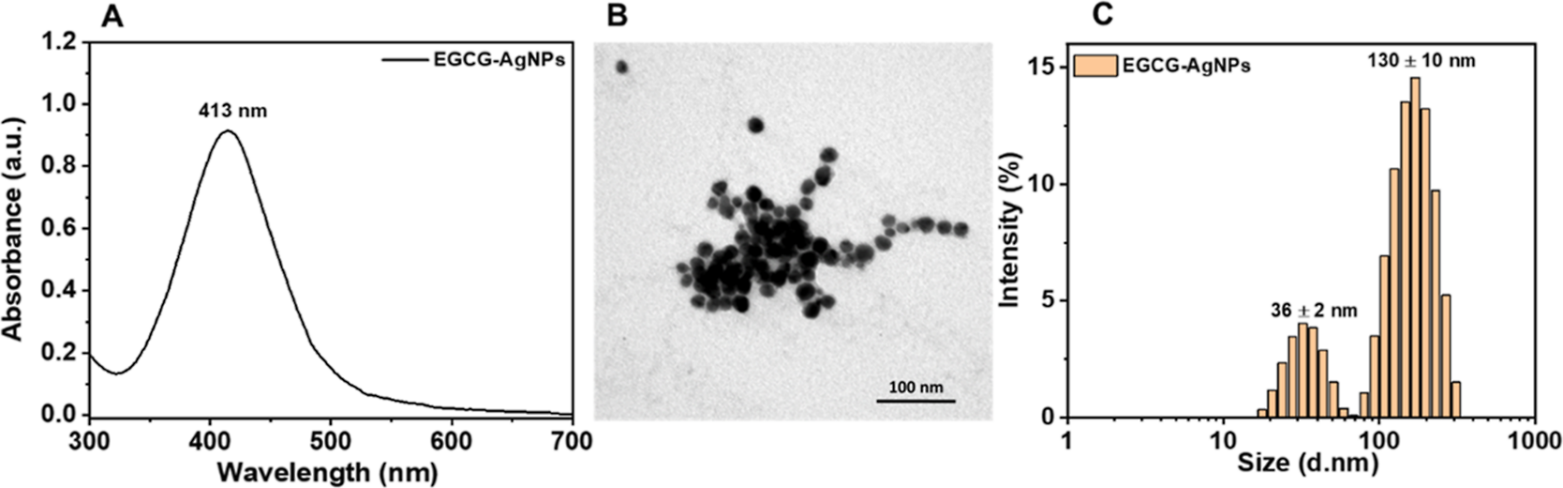
Characterization of EGCG-AgNPs. UV–visible spectrum (A), TEM image (B), and size distribution (C) of EGCG-AgNPs in water.

AgNPs were prepared by the reduction of Ag^+^ in a solution of silver nitrate (AgNO_3_) in the presence of EGCG, a polyphenol used as a reducing agent and an NP stabilizing agent due to the availability of hydroxyl (−OH) groups.^70,71^ The formation of AgNPs was confirmed by the occurrence of a UV–vis absorption band, peaking at 413 nm, which is characteristic of SPR (Figure 1A). TEM analysis showed spherical EGCG-AgNPs with a diameter of 14 ± 5 nm (Figure 1B), whereas dynamic light scattering (DLS) showed a hydrodynamic diameter (*d*_H_) of 36 ± 2 and 130 ± 10 nm (Figure 1C). The larger *d*_H_ values of the AgNPs (obtained with DLS) compared to those observed in the TEM images may be attributed to the formation of EGCG-AgNPs dimers (∼36 nm) or aggregates (∼130 nm). NP aggregates may form either due to surface aggregation processes as a result of poor coverage or weak binding of the ligand, or due to aggregation of the ligand itself at the surface of the NPs. The former would affect the SPR and result in a redshift in the absorbance; however, the relatively sharp absorbance band of the EGCG-AgNPs and the lack of an additional, redshifted peak implies that the population of larger NPs originates from surface aggregation of EGCG ligands rather than from aggregation of NPs. Figure S1A,B shows the time-dependent UV–vis spectra of EGCG-AgNPs in water and in phosphate-buffered saline (PBS) (pH 6.2), respectively. EGCG-AgNPs were stable in water, as indicated by the insignificant changes in the absorption band peak position and intensity in the course of 24 h (Figure S1A).

In contrast, adding the EGCG-AgNPs into PBS buffer resulted in a blueshift and a decrease in the intensity of the SPR absorption band (Figure S1B), indicating that AgNPs are unstable in PBS buffer.^72,73^ The reduced stability of EGCG-AgNPs in PBS relative to water may be explained by partial substitution of EGCG with salt, and it stands in agreement with previous reports of the aggregation of AgNPs upon the addition of salt.^74^ This was further supported by an increased negative ζ potential values of EGCG-AgNPs in PBS (−35 mV) when compared to water (−27 mV) (Figure S2). (d. nm) denoted the hydrodynamic diameter in nanometers.

### Interaction of Human Lysozyme with EGCG-AgNPs

**Figure 2:**
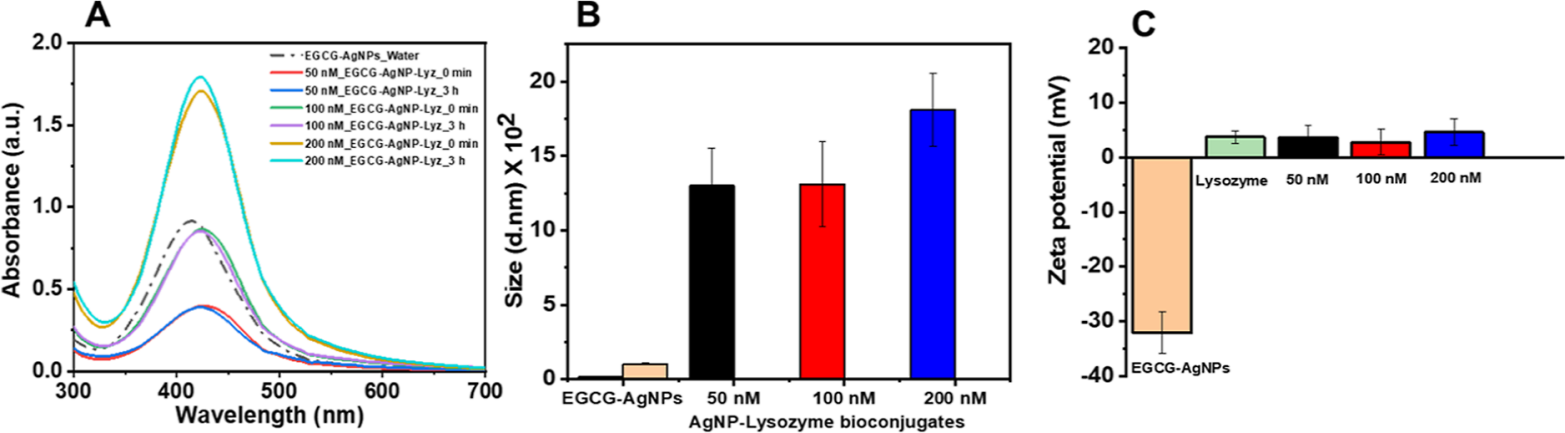
Characterization of EGCG-AgNP-lysozyme bioconjugates. (A) UV–visible spectra of EGCG-AgNP-lysozyme bioconjugates before and after 3 h incubation in PBS buffer (pH 6.2) at 37 °C. (B) Size (*d*_H_) of EGCG-AgNPs and their AgNP-lysozyme bioconjugates after 3 h incubation. (d. nm) denoted the hydrodynamic diameter in nanometers. (C) ζ potential of EGCG-AgNPs, lysozyme, and their corresponding AgNP-lysozyme bioconjugates at pH 6.2. The lysozyme concentration was fixed to 6.8 μM, and AgNP concentration was varied (50, 100, and 200 nM).

The interaction of lysozyme with EGCG-AgNPs in PBS (pH 6.2) was characterized by UV–vis, DLS, and ζ potential analyses. We fixed the concentration of lysozyme (6.8 μM) and added EGCG-AgNPs at increasing final concentrations. Addition of EGCG-AgNPs to a lysozyme/PBS buffer solution resulted in a redshift of the absorption band (Figure 2A). Interestingly, the peak position and the intensity of the SPR band remained unchanged even after incubation at 37 °C for 3 h. The redshift of the SPR absorption band is indicative of strong interactions between EGCG-AgNPs and lysozyme molecules, and it is also supported by an increase in the overall d_H_ for EGCG-AgNP-lysozyme bioconjugates (Figure 2B). However, the intensity of the SPR peak remained unchanged, suggesting that despite the increased size of the AgNP-lysozyme bioconjugates relative to EGCG-AgNPs, the AgNPs remained stable in solution. A possible explanation would be steric repulsion between lysozyme molecules. Increasing the lysozyme concentration (6.8, 20.4, and 34 μM) while keeping the EGCG-AgNPs concentration fixed (100 nM) did not affect the SPR absorption peak (Figure S3), indicating that a minimum lysozyme concentration (6.8 μM) is sufficient to form stable EGCG-AgNP-lysozyme bioconjugates, possibly due to saturation of the binding sites at the AgNP surface. In fact, measurement of the concentrations of lysozyme that was bound to EGCG-NPs and lysozyme that remained free in solution revealed that ∼20% of the lysozyme was bound to the NPs while 80% was unbound (see Experimental Section and Figure S4).

Interaction of EGCG-AgNPs with lysozyme was characterized by a positive surface charge, in agreement with the isoelectric point of lysozyme (10.7)^64−66^ being larger than the working pH (6.2). The positive ζ potential of the AgNPs confirmed lysozyme adsorption onto the EGCG-AgNPs and formation of EGCG-AgNP-lysozyme bioconjugates (Figure 2C). Importantly, despite the lower ζ potential (absolute value) of the EGCG-AgNP-lysozyme bioconjugates relative to EGCG-AgNPs, the EGCG-AgNP-lysozyme bioconjugates remained stable in solution, confirming further the steric stabilization of the NPs by lysozyme molecules in PBS buffer.

### Effect of EGCG-AgNPs on the Secondary Structure of Lysozyme

**Figure 3:**
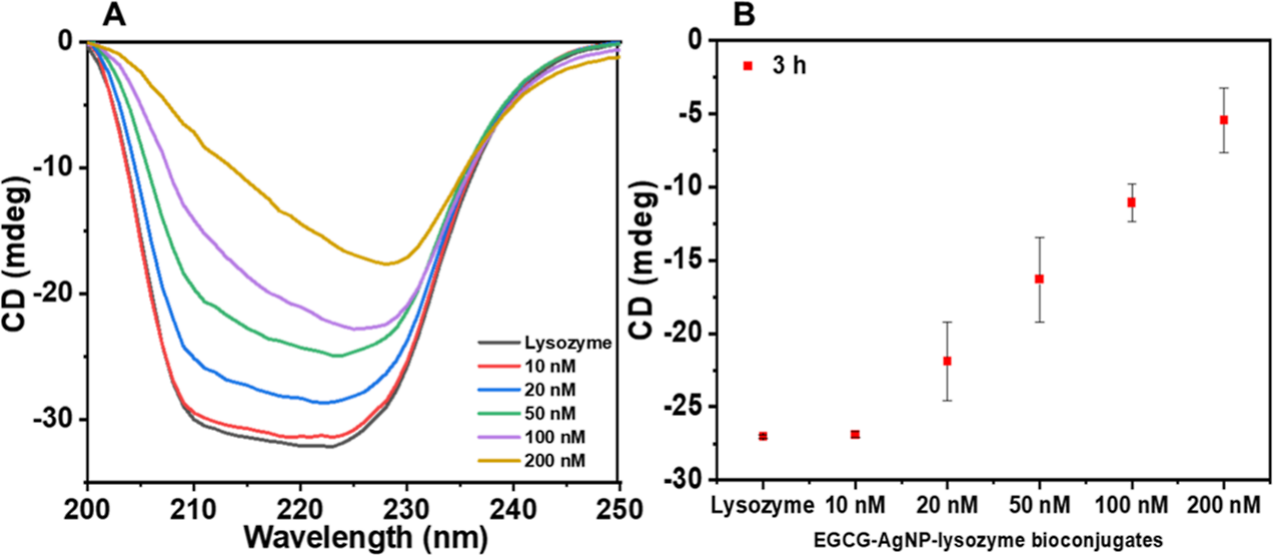
(A) CD spectra of free human lysozyme (6.8 μM) and in EGCG-AgNP-lysozyme bioconjugates with increased concentrations of AgNPs in PBS buffer (pH 6.2) after 3 h incubation at 37 °C. (B) Plot of ellipticity values at 208 nm for free lysozyme and EGCG-AgNP-lysozyme bioconjugates at pH 6.2.

Figure 3 shows the circular dichroism (CD) spectra of lysozyme alone and lysozyme with EGCG-AgNPs following 3 h incubation in varying concentrations, ranging between 10 and 200 nM. The CD spectra of lysozyme at pH 6.2 exhibit two negative minima at 208 and 222 nm, which is characteristic of α-helical content, corresponding to π–π* and *n*–π* transitions of the peptide bonds, respectively.^75^ Interestingly, adding EGCG-AgNPs to the lysozyme solution affected the intensity and shape of CD spectra in a concentration-dependent manner. Specifically, increasing the concentration of EGCG-AgNPs resulted in decreased ellipticity and transition of the lysozyme structure from α-helix to β-sheet at concentrations ≥100 nM. Loss of the characteristic alpha-helical peak at 208 nm upon the addition of EGCG-AgNPs is shown in Figure 3B. Table S1 shows relative fractions of secondary structures of lysozyme calculated by BeStSel software by best fitting the CD spectra with linear combinations of spectra of known protein structures.^76^ This analysis confirms the qualitative transition of lysozyme from being α-helix- to β-sheet-rich, quantifying the reduction in the α-helical fraction of the lysozyme from 20.5 to 3.4% in the presence of EGCG-AgNPs (200 nM) after 3 h incubation. Intramolecular hydrogen bonds stabilize lysozyme molecules and lead to an α-helical structure. Hydrogen bonding between hydroxyl-EGCG and lysozyme may interfere with the lysozyme intramolecular interaction and stand responsible for the structural changes of lysozyme.

### Effect of EGCG-AgNPs and EGCG-AgNP-Lysozyme Bioconjugates on *B. subtilis* Bacterial Growth and Biofilm Formation

**Figure 4:**
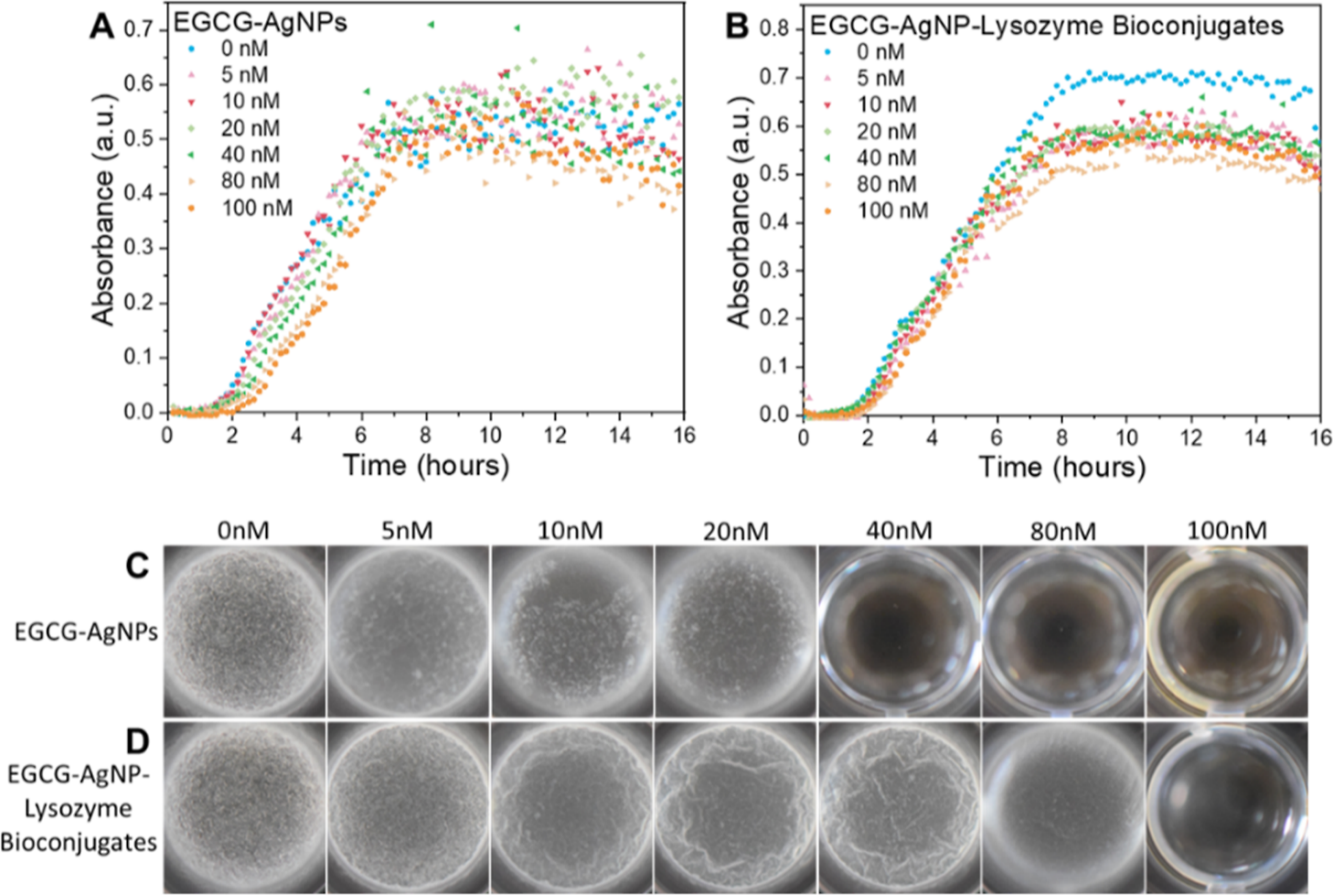
Effect of EGCG-AgNPs and their lysozyme bioconjugates on WT *B. subtilis* growth curves and biofilm assays. Growth curves in the presence of (A) EGCG-AgNPs and (B) EGCG-AgNP-lysozyme bioconjugates in PBS at pH 6.2. The concentration of the NPs is specified in the legends. Biofilm formation by *B. subtilis* in the absence and presence of increasing concentrations of EGCG-AgNPs (C) and EGCG-AgNP-lysozyme bioconjugates (D).

We examined the effect of the EGCG-AgNPs and EGCG-AgNP-lysozyme bioconjugates on cell viability and biofilm formation of WT *B. subtilis*. The growth curves were similar in the presence and absence of EGCG-AgNPs (Figure 4A, log/semi-log plot shown in Figure S5A), suggesting that EGCG-AgNPs had no antibacterial activity on planktonic *B. subtilis* cells. This result is surprising in light of the antibacterial activity of both free EGCG and free AgNPs.^77^ We speculate that the functional groups of EGCG attach to AgNPs, and therefore, EGCG loses its antibiotic activity. At the same time, EGCG binding to AgNPs prevents the leaching of Ag^+^ ions from the NPs, which, in turn, compromises nanoparticle antibiotic activity.

In contrast to the compromised antibacterial activity against cells in cultures, EGCG-AgNPs inhibited *B. subtilis* in biofilm-forming conditions at concentrations larger than 40 nM. Inhibition of biofilm formation could be attributed to a biocidal effect of EGCG-AgNPs; however, the growth curves in Figure 4A rule out this possibility because they remained unchanged even in the presence of EGCG-AgNPs at a 100 nM concentration. Therefore, EGCG-AgNPs may have interfered with biofilm formation pathways, such as the expression of ECM components or prevention of their proper assembly into a network,^8^ as well as communication by quorum sensing.^78^

Similarly to EGCG-AgNPs, when these NPs were additionally functionalized with lysozyme, they showed no biocidal activity on *B. subtilis* cells in the concentration range we have used (Figure 4B, log/semi-log plot shown in Figure S5B). However, the lysozyme corona reduced the effect of EGCG-AgNPs on *B. subtilis* biofilms. Specifically, biofilm inhibition occurred at concentrations larger than 80 nM EGCG-AgNP-lysozyme bioconjugates, which is larger than the 40 nM inhibitory concentration for biofilm inhibition by EGCG-AgNPs. The decrease in biofilm inhibition by the EGCG-AgNP-lysozyme bioconjugates may be either due to the blockage of negatively charged EGCG moieties by lysozyme or due to the loss of lytic activity of lysozyme against bacterial cell wall when it is bound to EGCG-AgNPs.

### Cytotoxicity of EGCG-AgNPs and EGCG-AgNP-Lysozyme Bioconjugates

**Figure 5:**
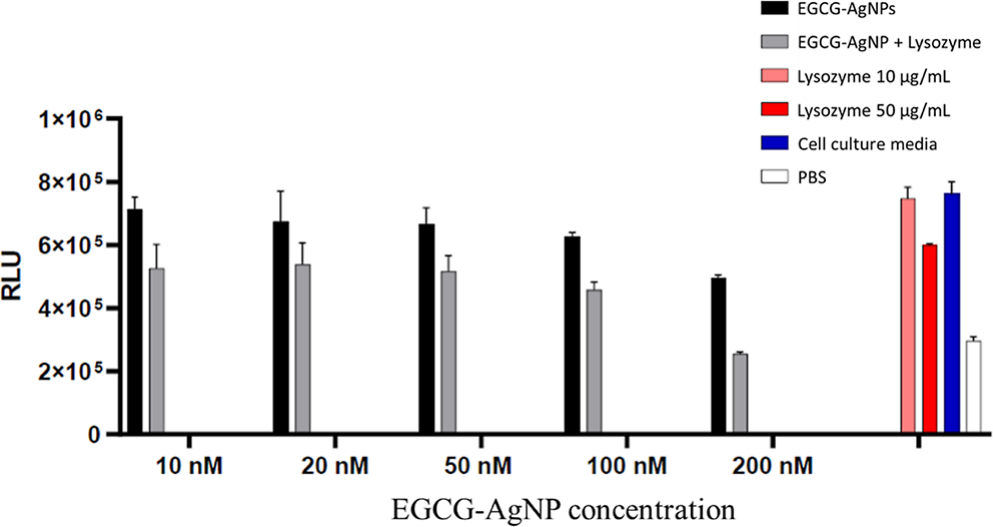
Cytotoxicity assay of HUVEC cells for different concentrations of EGCG-AgNPs (10, 20, 50, 100, and 200 nM) and the corresponding AgNP-lysozyme bioconjugates in endothelial cell culture media supplemented with fetal bovine serum (FBS). Measurement data was normalized as explained in the methods section.

We have further investigated the toxicity of EGCG-AgNPs and EGCG-AgNP-lysozyme bioconjugates on human umbilical vein endothelial cells (HUVEC) cells as a model system for endothelial cells, which form the inner layer of blood vessels. Figure 5 shows the HUVEC cell viability data after treatment with different concentrations of EGCG-AgNPs and EGCG-AgNP-lysozyme bioconjugates. EGCG-AgNPs are barely cytotoxic below 200 nM; however, a decrease in cell viability was observed at 200 nM EGCG-AgNPs. EGCG-AgNPs present a lower cytotoxicity when compared to EGCG-AgNP-lysozyme bioconjugates, the latter showing decreased cell viability already at 10 nM. The differences between the cytotoxicity of EGCG-AgNPs and lysozyme-functionalized EGCG-AgNPs may be related to faster uptake rates of the lysozyme bioconjugates by the cells. An additional possibility would be that human cells are more affected by structural changes of lysozyme, in particular of β-sheet structures, relative to bacterial cells.

## Conclusions

We have synthesized EGCG-AgNPs and studied their interaction with lysozyme. Conjugation of EGCG-AgNPs with lysozyme increased the stability of these NPs in PBS, and it induced structural changes in lysozyme from a dominant α-helical to a dominant β-sheet fraction in a concentration-dependent manner.

Studies of the inhibitory effect of EGCG-AgNPs and lysozyme-conjugated EGCG-AgNPs on Gram-positive wildtype *B. subtilis* biofilms showed that EGCG-AgNPs inhibited the formation of *B. subtilis* biofilm above 40 nM, whereas the lysozyme bioconjugates of EGCG-AgNPs were less effective and inhibited biofilms at higher concentration (≥80 nM). Interestingly, EGCG-AgNPs showed lower cytotoxicity when compared to EGCG-AgNP-lysozyme bioconjugates, possibly due to lower uptake propensity. Strikingly, both EGCG-AgNPs and lysozyme conjugated EGCG-AgNPs did not affect the viability of bacterial cells, suggesting that (i) bound EGCG is not harmful to bacterial cells, and (ii) NPs need to be uptaken into cells in order to cause damage.

Our results demonstrate that EGCG-AgNPs can be used as an antibiofilm agent against *B. subtilis* as they showed lower toxicity against human cells and a significant inhibitory effect on biofilm formation.

## Experimental Section

### Materials

Human lysozyme, (−)-EGCG, PBS, and silver nitrate (AgNO_3_) were purchased from Sigma-Aldrich (Taufkirchen, Germany). EtOH, HCl, and NaOH were purchased from Roth (Karlsruhe, Germany). The concentration of lysozyme was measured spectrophotometrically using a molar extinction coefficient of 38,940 mol^–1^ cm^–1^ at 280 nm. The water used was purified through an ultrapure water system, Millipore system Sartorius Stedim Biotech (Göttingen, Germany).

PBS was prepared by dissolving 0.137 M NaCl, 0.0027 M KCl, 0.01 M Na_2_HPO_4_, and 0.0018 M KH_2_PO_4_ in triple-distilled water and adjusting for pH with HCl. Except for NaCl, all chemicals were purchased from Sigma-Aldrich (Darmstadt, Germany). NaCl was purchased from J.T. Baker-Avantor (Center Valley, PA, USA). The water was purified through a Barnstead GenPure water purification system (Thermo Scientific).

### Epigallocatechin Gallate (EGCG)-AgNPs

EGCG (10 mM) solution was prepared by dissolving 4.58 mg of EGCG in 1 mL of deionized water, which was preadjusted to pH 8, and then it was added dropwise to 30 mL of 1 mM AgNO_3_ (5.03 mg in 30 mL water) solution with continuous stirring at 27 °C for 2 h. AgNO_3_ solution turned from colorless to yellowish brown upon addition of EGCG solution.

### EGCG-AgNP-Lysozyme Interaction

EGCG-AgNPs were added to lysozyme in PBS (pH 6.2), and the mixture was incubated at 37 °C for 3 h prior to characterization. For the analysis of protein structural changes, a fixed concentration of lysozyme (100 μg/mL) was mixed with various concentrations of EGCG-AgNPs (10, 20, 50, 100, and 200 nM) and then incubated at 37 °C for 3 h. Similarly, various concentrations of lysozyme (100, 300, and 500 μg/mL) were used for UV–vis analyses.

### EGCG-AgNP-Lysozyme Bioconjugates

EGCG-AgNPs (1 μM stock solution) were added to PBS buffer (pH 6.2) containing 100 μg/mL lysozyme and incubated at 37 °C for 4 h. EGCG-AgNP-lysozyme bioconjugates were obtained by centrifuging the above mixture at 17,000*g* at room temperature for 30 min and resuspending in PBS buffer. The bioconjugates were diluted with PBS buffer to various concentrations (5, 10, 20, 50, 80, and 100 nM).

### Determination of the Lysozyme Concentration on the AgNP Surface

The protein concentration on the EGCG-NPs and the unbound protein that remained in solution was determined by measuring absorbance at 280 nm (*A*_280_) using a DeNovix DS-11 FX+ spectrophotometer. A lysozyme calibration curve was prepared by measuring the *A*_280_ of a series of lysozyme dilutions of known concentrations (1–0.0156 mg/mL). The protein solution of lysozyme (100 μL of 68 μM) was mixed with an aqueous dispersion of EGCG-AgNPs (100 nM) to a final concentration of 6.8 μM. The EGCG-Ag-lysozyme mixture was incubated for 3 h at 37 °C, and the EGCG-NP-lysozyme bioconjugates were precipitated by centrifugation (17 000g × 30 min). The unbound (supernatant) protein was removed from the AgNP-lysozyme mixture, and the EGCG-AgNP-lysozyme bioconjugates pellet was resuspended in PBS. The absorbance of the supernatant and pellet was measured, and the lysozyme concentration was determined by plugging in the background subtracted absorbance in the absorbance versus concentration calibration curve (see Figure S4 for the calibration curve and lysozyme concentration determination).

### Circular Dichroism Spectroscopy Measurements

The far-UV CD spectra of lysozyme after incubation with AgNPs were recorded on a Chirascan spectrophotometer (Applied Photophysics, Leatherhead, UK). The spectrophotometer was purged with nitrogen gas before the experiments. Measurements were scanned between 200 and 250 nm, with an average of 5 scans using a 5 mm path length cuvette (Hellma Analytics, Müllheim, Germany). All spectra were measured at room temperature with a bandwidth of 1.0 nm. The final data were obtained by subtracting the buffer contribution from the original protein spectra. The fractional contents of the secondary structures were calculated by BeStSel software.^75^

### UV–vis Absorption Spectroscopy

A DeNovix DS-11 FX+ spectrophotometer (Biozym Scientific GmbH, Germany) was used to obtain the absorption spectra for EGCG-AgNPs, and the same after interacting with lysozyme. All the samples were measured between 200 and 850 nm using a 10 mm path length cuvette at room temperature.

### DLS and ζ Potential Measurements

Hydrodynamic diameter (*d*_H_) and ζ potential for EGCG-AgNPs and the corresponding lysozyme bioconjugates were determined using Zetasizer Ultra (Malvern Instruments, Kassel, Germany). Except lysozyme bioconjugates, all the samples were prepared as described above and filtered through a 0.2 μm filter. Before measurements, samples were equilibrated for 10 min at room temperature. Each size measurement was recorded, allowing 20 runs per measurement with a run duration of 5 s. An average of five separate measurements was used to assess the size of the samples. DTS1070 cells were used to measure the ζ potential of AgNPs and their lysozyme bioconjugates. A voltage of 180/40 V was used for the samples measured in deionized distilled water and PBS, respectively. All measurements were acquired at room temperature with an equilibration time of 5 min (20 runs per each measurement) between each measurement. The reported ζ potential is an average of five independent measurements.

### Transmission Electron Microscopy

The flotation method was used for the negative staining procedure. AgNPs were allowed to adsorb onto a glow-discharged Pioloform carbon-coated 400-mesh grid for 5 min. The grid was then transferred onto two droplets of deionized water and finally onto a drop of 1% aqueous uranyl acetate for 30 s. After blotting with filter paper and air-drying, the samples were examined with a transmission electron microscope (LEO 906, Carl Zeiss Microscopy Deutschland GmbH, Oberkochen, Germany) at an acceleration voltage of 80 kV. For image acquisition, a wide-angle dual speed CCD camera (SharpEye, Tröndle, Moorenweis, Germany) was used, operated by ImageSP software. All micrographs were edited by using Adobe Photoshop CS6.

### Biofilm and Growth Curve Assays

Liquid culture of WT *B. subtilis* was grown in LB broth at 37 °C for 16 h. For the biofilm formation assay, 2 μL of starting culture was added to 88 μL of MSgg medium in a 96-well plate and incubated (24 h, 30 °C) with 10 μL of EGCG-AgNPs or with EGCG-AgNP-lysozyme bioconjugates to achieve various final concentrations: 5, 10, 20, 40, 80, and 100 nM. Biofilms were captured with a Nikon D3300 camera with a micro Nikkor 85 mm lens. For the growth curve assay, 2 μL of starting culture were added to 88 μL of LB broth in a 96-well plate with 10 μL of EGCG-AgNPs orEGCG-AgNP-Lysozyme bioconjugates in various final concentrations, as specified above for biofilms. Bacterial growth was measured in a microplate reader (Tecan Spark 10M, Tecan Trading AG, Switzerland) for 16 h at 37 °C with 180 rpm orbital shaking.

EGCG-AgNPs stock solutions were prepared in triple-distilled water, and EGCG-AgNP-lysozyme stock solutions were prepared in PBS buffer. Control experiments without EGCG-AgNPs were performed with water as a substitute of EGCG-AgNPs and PBS buffer as substitute for EGCG-AgNP-lysozyme bioconjugates.

### Cell Viability Assay

Cell viability assays were carried out following manufacturer’s instructions from the CellTiter-Glo 2.0 assay from Promega (Madison, USA). In brief, 5 × 10^4^ HUVEC/mL were seeded in endothelial cell growth medium MV (PromoCell, Heidelberg, Germany) with SupplementMix. Cells were incubated for 3 h in an opaque 96-well plate at 37 °C and 5% CO_2_. Then, media was removed, and different concentrations of AgNPs were added, as indicated in the respective graphs. After 24 h incubation at 37 °C and 5% CO_2_, 100 μL of CellTiter-Glo 2.0 was added to each well, and the luminescence signal was measured in a Cytation 5 imaging reader (BioTek Instruments Inc., Winooski, USA) after 10 min incubation.

## Supporting Information Available

UV–vis absorption spectra of EGCG-AgNPs in water and PBS; ζ potential of EGCG-AgNPs in water and PBS; UV–visible spectra of EGCG-AgNP-lysozyme bioconjugates; determination of the EGCG-NPs-bound and unbound lysozyme fraction; growth curves of WT *B. subtilis* in the presence of EGCG-AgNPs and EGCG-AgNP-lysozyme bioconjugates; percentages of secondary structures of lysozyme in the presence of EGCG-AgNPs at pH 6.2 (PDF)
